# Postoperative clinical nursing care pathway for patients with mucormycosis: An experience from a tertiary care hospital in Rajasthan, India

**DOI:** 10.3126/nje.v11i4.40498

**Published:** 2021-12-31

**Authors:** Nipin Kalal, Suresh K Sharma, Kapil Soni

**Affiliations:** 1,2 College of Nursing, All India Institute of Medical Sciences Jodhpur, India; 3 Otorhinolaryngology, All India Institute of Medical Sciences Jodhpur, India

**Keywords:** Clinical pathway, COVID-19, Nursing care, Postoperative care, Postoperative recovery assessment

## Abstract

**Background:**

The COVID-19 pandemic is a serious global health threat and it has numerous impacts on human life. India faced the problem of the second wave of COVID-19 and an unexpected new predicament in the form of mucormycosis has been added. The use of steroids drugs for long duration and comorbidity with COVID-19 infections are the risk factors of mucormycosis. It is important to understand the postoperative clinical pathway to assess and determine the policy and protocol, which help patients fasten their recovery, prevent further complications and readmission.

**Methods:**

A cross-sectional descriptive design was used to conduct the study. We adopted the validated Immediate Post-Operative Recovery Assessment (IPR-PA) Scale to assess the postoperative clinical nursing care pathway for patients with mucormycosis.

**Results:**

The current study shows that patients had highest score in the physiology clinical recovery domain (75.25%) and the lowest post-operative clinical recovery score in psycho-social domain (20.83%). There was a significant positive correlation was found between all the domains. The medication status domains found significantly associated with participants’ age (P=.021) and physiological domains has shown significant association with received oxygen therapy during hospitalization (P=.046).

**Conclusion:**

Postoperative clinical nursing care pathway was effective to determine the progress of a patient. It helps us to know the parameter of different domains namely being physiological, physical, psycho-social and medication status. Patients required psycho-social support due to the epidemic and fear from disease.

## Introduction

India had a painful and bitter experience because of the devastating second wave of COVID-19 with an increase mortality rate as compared to the first wave [1]. India is belligerent against a rapid increase in Covid-19 cases, but a revolting and unusual fungal illness known as Mucormycosis (black fungus) has emerged in patients suffering from or recovering from COVID-19 [2]. During the second wave of COVID-19, many associated health issues are encountered, in which one such disease being Mucormycosis, was becoming a challenging issue in India due to its unprecedented surge and high morbidity. The increased number of mucormycosis in COVID-19 patients are prevalent primarily because of the increased use of steroids like dexamethasone, especially among diabetics [3]. Many patients with mucormycosis require immediate surgical intervention to prevent infection to the adjacent organs. Healthcare has many challenges to develop the new intervention to measure the outcome of a postoperative clinical pathway. Many innovative and healthy plans are required to become the more skilful observers and competent in quality care. The Enhanced Recovery after Surgery (ERAS) program is focused on evidence-based perioperative care and Scientifics changes required in routine clinical practice [4].

A study carried out by Muller MK, et al. reported that Clinical pathways reduced the postoperative hospital stay by 28% from a mean of 6.1 to 4.4 days. This study concludes that clinical pathways not only benefit clinically and economically but also help in reduce the use of resources, without any undesirable influence on complications or re-hospitalization [5]. Nursing care pathway is a symbiotic model which enables the visibility of the nurse as well as improves the satisfaction among patients. By following this pathway, the nurses take accountability for patient care by implementing balanced technological input and determined humanistic care [6].

The clinical pathway on postoperative recovery patient following pancreaticoduodenectomy was done and results suggested the CPW reduced inconsistency and permitted a more substantial proportion of patients to receive all the fundamentals of care, ensuing in improved excellence and efficiency of care based on recent best evidence endorsements [7].

Heeba M, conducted a study to investigate the effect of clinical pathways of postoperative nursing care for patients undergoing gynaecologic operations on the postoperative outcome. The study shows that the clinical pathway was effective in improving postoperative outcomes related to early ambulation, early oral intake, bowel mobility, hours to remove catheters and drains, a short length of stay and patient satisfaction [8].

The Post-operative outcome of any procedure depends on the quality of nursing care renders to the patients. It is essential to monitor the clinical pathway for early detection of immediate complications and discomfort of the patient due to surgical intervention. The Post-operative clinical nursing pathway will be beneficial in efficient management, shorter length of stay and improving the patient’s condition. Until now no such study is conducted in India and also hope and believed that study finding will help and contribute to safer practices which may eventually result in positive progressive postoperative recovery among such patients.

This study will investigate the postoperative clinical nursing pathway of patients diagnosed with mucormycosis. Furthermore, it will help in improving patient’s condition by efficient management of postoperative care and shorter lengths of hospital stay.

## Methodology

### Study design and the participants

A cross-sectional descriptive design was used to assess the post-operative clinical nursing pathway of patients with mucormycosis. The total enumeration sampling technique was used to enrol the patients who visited the hospital from May to July 2021 at tertiary care hospitals in Rajasthan, India

Questionnaire design and validation:

Sociodemographic data and Immediate post-operative assessment scale (IPR-PA) were used to collect the patient’s data. The details of the questionnaires used in the study are as follows:

### Sociodemographic sheet

This sheet was used to collect demographic information like age, gender, occupation, education, marital status and residence. Clinical information is also collected like the previous history of COVID-19 positive, admitted in hospital with duration, treatment therapy such as the use of steroids and oxygenation during admission in hospital, the status of vaccination and comorbidity. The profile sheet sought validation from an expert consultant in the field of medicine, ENT, Dental and nursing and it was pretested in eligible patients before final use.

Immediate post-operative assessment scale (IPR-PA)

IPR-PA was validated by experts and found to be 80% reliable with Cronbach’s α = 0.81. The content validity ratio (CVR) for scale was 0.89, showed an acceptable level of internal consistency of the scale. This scale consists of 4 domains namely physiological, physical, psycho-social and medication status. The domain of physiology consists of 10 items such as pulse, blood pressure, temperature, respiration, oxygen saturation, pain, consciousness, wound healing, nausea/vomiting and sleep disturbance. The domain of physical has 05 items that are feeding, elimination-bowel, elimination-bladder, mobility and personal hygiene. The domain on psycho-social has 03 items that are worry and anxiety, social isolation and discomfort. Two items are under the domain of medical status for the administration of anti-microbial and analgesic drugs. All the given parameters were monitored post-operatively for 7 days and scores are given as per the data received from the patients. A higher scores represent the positive recovery and a lower score interpretate the poor recovery.

### Data collection

Data were obtained by monitored the post-operative parameters of the patients for 7 days in Mucormycosis ward using a structured validated scale by trained nursing officers with help of author.

### Inclusion criteria

All the patients who were operated for mucormycosis and provided informed consent for voluntary participation in the study were included. Patients who remain admitted in hospital for 7 days after surgical intervention were eligible to participate in our study.

### Sample size calculation

A sample size calculation was based on the finite population by using Slovin’s formula, n=N/(1+Ne2); N= Total number of operated cases (134) & e = Margin of error, that is 0.05, confidence interval as 95 percent, and minimum estimated sample size needed is 82. After considering drop out of 10 % approximately 90 subjects were enrolled in the study.

### Outcome Variable

The main outcome variables of the study were to investigate the postoperative clinical nursing pathway of patients diagnosed with mucormycosis.

### Explanatory variable

Socio-demographics variables such as age, gender, place of living and Clinical variables consist of previous history of COVID-19 positive, admitted in hospital with duration, treatment therapy such as the use of steroids and oxygenation during admission in hospital, the status of vaccination and comorbidity.

### Ethical committee approval

Ethical clearance was obtained from the Institutional Ethics Committee wide letter no. AIIMS/IEC/2021/3623. It was ensured & declared that confidentiality and anonymity of the subjects and the data collected will be maintained throughout the study.

### Data management and statistical analysis

Data were coded and transferred in an excel sheet and statistical package for the social sciences (SPSS 27) for statistical analysis. Descriptive statistics of the participants’ demographic and clinical data were provided as frequency and percentage for categorical variables. For all analyses, P 0.05 was considered as statistically significant. All statistical analyses were performed by using the SPSS version 27.

## Results

The demographic details of the included patients (N=90) are shown in Table 1. Almost half of the patients 46 (51.1%) were aged less than 50 years and 44 (48.9%) were aged more than 50 year. Of the participants, 57(63.3%) were male and 33 (36.7%) were female. Approximately half 46 (51.1%) of the patients were employed. In terms of education, 35 (38.9%) of patients studied up to primary and 21 (23.3%) were illiterate. Most of the patients 89 (98.9%) were married and the majority of them 59 (65.6%) were living in rural areas. A greater number of patients 71 (78.9%) had a history of COVID-19 infection, out of them 53 (58.9%) patients were admitted in hospital and about 33 (36.7%) of them reported that they received oxygen therapy during hospitalization. A higher proportion of the patients 65 (72.2%) had received steroids as a treatment of COVID-19 infection. Overall, 32 (35.56%) of patients were vaccinated, among only 13 (14.44%) patients have received both the doses. The major co-morbidities were diabetes mellitus in 46 (51.11%) patients followed by hypertension 11(12.22%).

Table 2 depicts day-wise mean score of clinical recovery domains which shows positive trends as the days passed. Hence it is showing the positive recovery of patients.

Table 3 depicts the domain wise mean and SD of post-operative clinical recovery score. It is found that mucormycosis patients had highest score in the physiology clinical recovery domain (75.25%) followed by the physical domain (74.60%). Mucormycosis patients had the lowest post-operative clinical recovery score in psycho-social domain (20.83%). Hence, the psycho-social domains need supportive intervention to fasten the recovery and better progress.

A significant positive correlation was found between physiological, physical, psychosocial and medication status domains. (Table:4)

Table 5 shows the association of clinical recovery domains with demographic variables among participants. The medication status domains found significantly associated with participants’ age (P=.021) and physiological domains has shown significant association with received oxygen therapy during hospitalization (P=0.46). However, the rest of clinical recovery domain score did not show any significant association to participants’ demographic variables.

## Discussion

Our study findings are unique as we have included mucormycosis patient and post-operative nursing care path way has been monitored. As per literature no such study has been conducted on mucormycosis and not even on any other disease condition. This study finding clearly state that nurses play a vital role in the recovery of patients. Such studies not only help to assess the patient recovery but also help to prepare the post-operative protocols and unique strategies to address physiological and psychological issues and challenges of the patients.

### Mucormycosis and COVID-19 infections & vaccinations

In this preliminary finding a greater number of patients, 71 (78.9%) had a history of COVID-19 who were reported with mucormycosis. These findings are alarming and should be taken seriously to prevent fungal infection as a part of post COVID-19 complications. In this study, about more than half of the participants 57(63.3%) were males who had been admitted with mucormycosis, Similar findings have been reported for other related studies which found that 47 (67%) were males with the same complaints [9]. Furthermore, these findings also have particular importance considering that only 32 (35.56%) of patients were vaccinated.

### Mucormycosis and it’s Risk factors

Our study findings clearly indicate that 51.11% of patients having comorbidity of diabetes mellitus who are reported with mucormycosis. Our study results were somewhere in line with another study done by Kumari A, et al. [10] reported 80% cases of diabetes mellitus associated with mucormycosis. Our findings indicated that a significant number of patients (72.2%) had received steroids for COVID-19. These findings were similar to previous studies, which reported that (84.6%) patients had received prior steroids for COVID-19[11]. Furthermore, our study revealed that 37% of the patient received oxygen therapy during hospitalization as a part of treatment for COVID-19 infection, which is very significant with the finding of another study where 57% of patient needed oxygen support for COVID-19 infection [12]. Agnihotri, et al. reported that a significant increase in the COVID-19 associated mucormycosis cases is strongly associated with treatment of corticosteroid in the diabetic patients treated for COVID-19. This finding is congruent to our study where 72.2% patients received steroids as a part of treatment for Covid-19 infection during hospitalization [13].

### Postoperative clinical pathway

On the contrary Tremblay St-Germain A, et al. [7] concluded that patients were achieved clinical pathway goals and outcomes, were positive which were comparable to our study findings. The current study result shows that medication status scores 2.15 which indicate most of the patient received antifungal as a part of treatment. These findings are in resemblance with another similar study [9]. According to a study conducted by Sharma S, et al. the prognosis remains poor even with aggressive surgery and intravenous anti-fungal therapy [14] but surprisingly our study findings suggest that patient had a positive post-operative clinical recovery scores. The present study is compared to a study done by Kathrin Rothe, et al. who found the poor physiological status of these patients [15]. Al-Tawfiq JA, et al. demonstrated that the outcome was favourable for patients who had surgical debridement. The result of our study are consistent that the patients who had surgical intervention had positive outcome [16]. Therefore, early diagnosis, surgical intervention and therapeutic postoperative nursing management is necessary for the patient to recover from mucormycosis.

## Limitation of the study

This study has certain limitations. First, the study was conducted in a single centre. The patient’s data were collected for seven days after surgery, and the outcome is not evaluated. The patient’s recovery was dependent on several factors, so generalization of findings was not possible.

## Conclusion

The postoperative clinical nursing care pathway for the patients who had undergone the surgical intervention for mucormycosis revealed that all the patients were having positive trends and the items under domains are progressively moving in a positive direction. Patients need psycho-social support because of epidemic and COVID-19 infection.

Therefore, it is concluded that postoperative clinical nursing care pathway helps us to identify the various parameter related to patient progress and we can use effective interventions to overcome with the huddles.

### Future scope of the study

Preliminary findings recommend the training programs for nurses in order to enhance their knowledge and skills regarding clinical pathway management. The same study can be done by using a different protocols of management and evidence based practices, with a large sample size and in a different settings.

### What is already known on this topic?

Mucormycosis is mainly associated with COVID-19 patients who has received steroids and having comorbidity like diabetes mellitus. All the patients has anxious feeling towards disease condition.

### What this study adds

This was a modest study to understand the post-operative nursing clinical pathway and disease progress. This study reports the lowest psycho-social score among patients who need support from nursing staffs and physicians. Till now no such study is conducted in India so such study may help to know the problems encountered by the patients in postoperative phase and how the nurse will act to such problems. Such scientific study has not done before for our population and setting and we hope, this will flag the way for a better-quality prospective study in future which may signpost need for a further policy and protocols.

## Figures and Tables

**Table 1:** Frequency and percentage distribution of demographic variables (n = 90)

Variables	Frequency (%)	
**Age (Year)**		
**≤50**	46 (51.1)	
**≥50**	44 (48.9)	
**Gender**		
**Male**	57 (63.3)	
**Female**	33 (36.7)	
**Occupation**		
**Unemployed**	46 (51.1)	
**Employed**	44 (48.9)	
**Education level**		
**Illiterate**	21 (23.3)	
**Primary education**	35 (38.9)	
**Secondary education and above**	34 (37.8)	
**Marital status**		
**Single**	01 (1.1)	
**Married**	89 (98.9)	
**Place of living**		
**Urban**	31 (34.4)	
**Rural**	59 (65.6)	
**History of COVID-19 infection**		
**Yes**	71 (78.9)	
**No**	19 (21.1)	
**Hospitalization due to COVID-19**		
**Yes**	53 (58.9)	
**No**	37 (41.1)	
**Received steroids treatment for COVID-19 infection**		
**Yes**	65 (72.2)	
**No**	25 (27.8)	
**Received oxygen therapy during hospitalization**		
**Yes**	33 (36.7)	
**No**	57 (63.3)	
**Status of vaccination**		
**Unvaccinated**	58 (64.44)	
**Vaccinated**	32 (35.56)	
**If yes then**	1^st^ Dose	2^nd^ Dose
**Covaxin**	2 (2.22)	3(3.33)
**Covishield**	17 (18.88)	10(11.11)
**Any comorbidities**		
**DM**	46 (51.11)	
**HTN**	11 (12.22)	
**HIV positive**	01 (1.11)	
**Renal disease**	01 (1.11)	

**Table 2:** Table 2: Day-wise mean score of participants

Clinical Recovery Domains	Day-1	Day-2	Day-3	Day-4	Day-5	Day-6	Day-7
**Physiological**	11.73	12.08	13.64	15.95	16.86	17.51	17.60
**Physical**	5.24	5.91	7.06	8.0	8.33	8.82	8.86
**Psycho-social**	0.60	0.68	0.91	1.33	1.71	1.77	1.77
**Medication status**	2.15	2.17	2.21	2.28	2.32	2.51	2.51

**Table 3:** Domain wise mean and standard deviation of participants

Clinical Recovery Domains	Score range	Mean±SD	Percentage
**Physiological**	0-20	15.05±2.34	75.25%
**Physical**	0-10	7.46±1.32	74.60%
**Psycho-social**	0-6	1.25±0.48	20.83%
**Medication status**	0-4	2.30±0.13	57.50%

**Table 4:** Correlation between the Domains

Correlations				
Domains	Physiological	Physical	Psychosocial	Medication status
**Physiological**		.987^**^	.993^**^	.909^**^
**Physical**	.987^**^		.970^**^	.892^**^
**Psychosocial**	.993^**^	.970^**^		.911^**^
**Medication status**	.909^**^	.892^**^	.911^**^	

**Table 5:** Association of Clinical Recovery Domains with demographic variables among participants

Variables	Physiological Mean (SD)	P value	Physical Mean (SD)	P value	Psycho-social Mean(SD)	P value	Medication status Mean (SD)	P value
**Age**								
**≤50**	17.39 ±2.76	.460	9.08±2.21	.394	1.91±2.66	.619	2.32±.70	.021*
**≥50**	17.81 ±2.68		8.63±2.75		1.63±2.59		2.70±.82	
**Gender**								
**Male**	17.85±2.29	.236	9.15±1.72	.146	1.92±2.67	.473	2.57±.82	.282
**Female**	17.15±3.31		8.36±3.40		1.51±2.55		2.39±.70	
**History of COVID-19 infection**								
**Yes**	17.38±2.79	.139	8.73±2.73	.326	1.69±2.56	.543	2.47±.79	.453
**No**	18.42±2.26		9.36±1.16		2.10±2.86		2.63±.76	
**Hospitalization due to COVID-19**								
**Yes**	17.58±2.58	.950	8.90±2.55	.860	2.11±2.75	.147	2.52±.82	.805
**No**	17.62±2.93		8.81±2.42		1.29±2.36		2.48±.73	
**Received steroids treatment for COVID-19 infection**								
**Yes**	17.32±2.87		8.61±2.82	.124	1.50±2.47	.116	2.43±.74	.117
**No**	18.32±2.13	.120	9.52±1.04		2.48±2.90		2.72±.84	
**Received oxygen therapy during hospitalization**								
**Yes**	16.90±3.24	.046*	8.66±2.72	.565	2.36±2.80	.107	2.66±.85	.152
**No**	18±2.29		8.98±2.36		1.43±2.47		2.42±.73	
**Status of vaccination**								
**Unvaccinated**	17.93±2.33	.120	8.96±2.34	.615	1.86±2.61	.684	2.44±.72	.308
**Vaccinated**	17±2.25		8.68±2.76		1.62±2.66		2.62±.87	

*Significant (p< 0.05)
